# Tumor-induced osteomalacia characterized by “painful knee joint with difficulty in moving”: a case report

**DOI:** 10.1186/s12902-022-01078-4

**Published:** 2022-07-08

**Authors:** Lan Jiang, Qing-Qing Tan, Chen-Lin Gao, Ling Xu, Jian-Hua Zhu, Pi-Jun Yan, Ying Miao, Qin Wan, Yong Xu

**Affiliations:** 1 Department of Endocrinology and Metabolism, The Affiliated Hospital of Southwest Medical University, Luzhou, 646000 Sichuan China; 2 Cardiovascular and Metabolic Diseases Key Laboratory of Luzhou, The Affiliated Hospital of Southwest Medical University, Luzhou, Sichuan P.R. China; 3 Sichuan Clinical Research Center for Nephropathy, The Affiliated Hospital of Southwest Medical University, Luzhou, Sichuan P.R. China; 4 Metabolic Vascular Disease Key Laboratory of Sichuan Province, The Affiliated Hospital of Southwest Medical University, Luzhou, Sichuan P.R. China

**Keywords:** Tumor-induced osteomalacia, Fibroblast growth factor-23, Hypophosphatemia, ^68^Ga DOTATATE PET/CT, Case report

## Abstract

**Background:**

Tumor-related osteomalacia (TIO) is a rare paraneoplastic syndrome characterized by severe hypophosphatemia and osteomalacia. The diagnosis of TIO can be very difficult because of its nonspecific nature of clinical manifestations. Here we reported a case of young TIO patient with “painful knee joint with difficulty in moving” to improve the clinical diagnosis and treatment levels.

**Case presentation:**

The patient’s clinical features were consistent with TIO. A tumor was successfully located in left tibial by ^68^Ga-DOTATATE PET/CT, and then was surgically resected. Upon pathologic assessment, the tumor was diagnosed as phosphaturia stromal tumor (PMT) with positive Vim staining. After the surgery, serum phosphate level rapidly recovered and symptoms significantly improved.

**Conclusion:**

TIO should be considered in patients with chronically hypophosphorus osteomalacia in the setting of no family history. Early removal of the responsible tumors is clinically essential for the treatment, and imaging examination is of great significance for tumor localization.

## Background

TIO is a rare paraneoplastic syndrome caused by tumors that ectopically overproduce fibroblast growth factor-23 (FGF-23), leading to decreased renal tubular reabsorption of phosphate. It is characterized by severe hypophosphatemia and osteomalacia [1]. Symptoms of TIO vary but mainly are gradually progressive bone pain and muscle weakness, pathological fracture and height shorten in severe cases [2]. It was first reported by Mc-cance in 1947, and less than 500 cases have been reported worldwide till now [3]. The diagnosis of TIO can be very difficult because of its insidious onset and nonspecific nature of clinical manifestations. The longer the course of the disease, the higher the disability rate [4]. Therefore, we described a TIO patient with “painful knee joint with difficulty in moving” as the typical manifestation and reviewed relevant literatures, in order to improve the recognition of this rare disease.

## Case presentation

### Case history

The 22-year-old female patient was admitted to the hospital on August 25, 2021 with the chief complaint of “double knee joints pain for 10+ years, aggravation for 2+ months, accompanied by groin pain for 2+ weeks”. 10+ years ago, she had recurrent pain in both knees without obvious inducement, occasionally with knee valgus and knee dislocation. 2+ years ago, she developed low back pain which was relieved after rest, but the spine moved freely. 1+ years ago, she experienced severe pain in her low back and loss of mobility. She needed help to get up and down stairs, usually could only walk slowly. 2+ months ago, the pain became progressively worse and she developed pain in the groin area and the right 12th rib after a tumble. MRI showed bone marrow edema in the right intertrochanteric, femoral neck, lower left femur, and tibial intercondylar crest. 2+ weeks ago, she had occasional wandering pain of joints all over the body, accompanied by difficulty in walking and increased pain after weight bearing. Biochemical tests revealed hypophosphatemia (0.47 mmol/L), high serum alkaline phosphatase (257 IU/L), low 25-OH Vit D (41.1 nmol/L), and dual-energy X-ray absorptiometry (DXA) demonstrated reduced bone density. Then she was admitted into the Department of Endocrinology of our hospital for further treatment. She had lost 3 cm in height over 10 years. Her medical history included polycystic ovary syndrome (PCOS) for nearly 3 years, with irregular menstruation. Deny bad eating habits, long-term absence from sunlight, chronic diarrhea and the history of taking adefovir dipivoxil or other drugs. No history of recurrent skin hemorrhages or tooth loss or fracture. No family history of skeletal, metabolic or hormonal disorders.

### Physical examination

She is 142 cm tall and weighs 46 kg, with no obvious positive signs in heart, lung and abdominal examination. Lower limbs are unequal in length and circumference (Relative length of lower limbs: R 77.5 cm, L 76 cm; absolute length of lower limbs: R 68 cm, L 67 cm. Thigh circumference: R 47.5 cm, L 45.5 cm; calf circumference: R 37.5 cm, L 33.5 cm) (Table 1). Physical examination reveales scoliosis deformity without tenderness and percussion pain in spinous process, hip flexion, extension, internal and external rotation (+), adduction, abduction (−), Moberg sign (+), Lasegue sign (−), both knee joints pain, limited movement, left knee joint evagination deformity, “X” shaped leg. No swelling, tenderness, dislocation of joints, no atrophy of muscles, no paralysis of limbs, no enhancement or weakening of muscle tension.

**Table 1:** Physical examination of the patient with tumor-induced osteomalacia (TIO)

Items	Relative length of lower limbs (cm)	Absolute length of lower limbs (cm)	Thigh circum-ference (cm)	Calf circum-ference (cm)
R	77.5	68	47.5	37.5
L	76	67	45.5	33.5

### Preoperative laboratory examination (august 25, 2021)

(1) Alkaline phosphatase: 270.4 U/L (n: 35-100 U/L); (2) Phosphorus: 0.52 mmol/L (n: 0.85–1.51 mmol/L); (3) Calcium: 2.29 mmol/L (n: 2.11–2.52 mmol/L); (4) Magnesium: 0.91 mmol/L (n: 0.75–1.02 mmol/L); (5) Creatinine: 42.9umol/L (n: 41-73umol/L); (6) Calcitonin: 15.3 pg/ml (n: 0.0–18.0 pg/ml); (7) Parathyroid hormone: 61.23 pg/ml (n: 8.70–79.60 pg/ml); (8) 25-OH Vit D: 16.00 ng/ml (deficiency: < 20 ng/ml); (9) Total procollagen I N terminal peptide: 90.84 ng/ml (n: premenopausal 8.53–64.32 ng/ml; Postmenopausal 21.32–112.80 ng/ml); (10) β-Crosslaps: 623.40 pg/ml (n: premenopausal 68.00–680.00 pg/ml; postmenopausal 131.00–900.00 pg/ml); (11) N-MID osteocalcin: 15.30 ng/ml (n: premenopausal 4.11–21.87 ng/ml; postmenopausal 8.87–29.05 ng/ml) (Table 2).

**Table 2:** Preoperative laboratory examination findings of the patient

Items	Result	Reference range
Alkaline phosphatase (U/L)	270.4	35–100
Phosphorus (mmol/L)	0.52	0.85–1.51
Calcium (mmol/L)	2.29	2.11–2.52
Magnesium (mmol/L)	0.91	0.75–1.02
Creatinine (umol/L)	42.9	41–73
Calcitonin (pg/ml)	15.3	0.0–18.0
Parathyroid hormone (pg/ml)	61.23	8.70–79.60
25-OH Vit D (ng/ml)	16.00	deficiency: < 20
Total PINP (ng/ml)	90.84	8.53–64.32
β-Crosslaps (pg/ml)	623.40	68.00–680.00;
N-MID osteocalcin (ng/ml)	15.30	4.11–21.87 ng/ml;

*PINP* procollagen I N terminal peptide

### Preoperative imaging examination

#### CT (August 30, 2021)

**Fig. 1:**
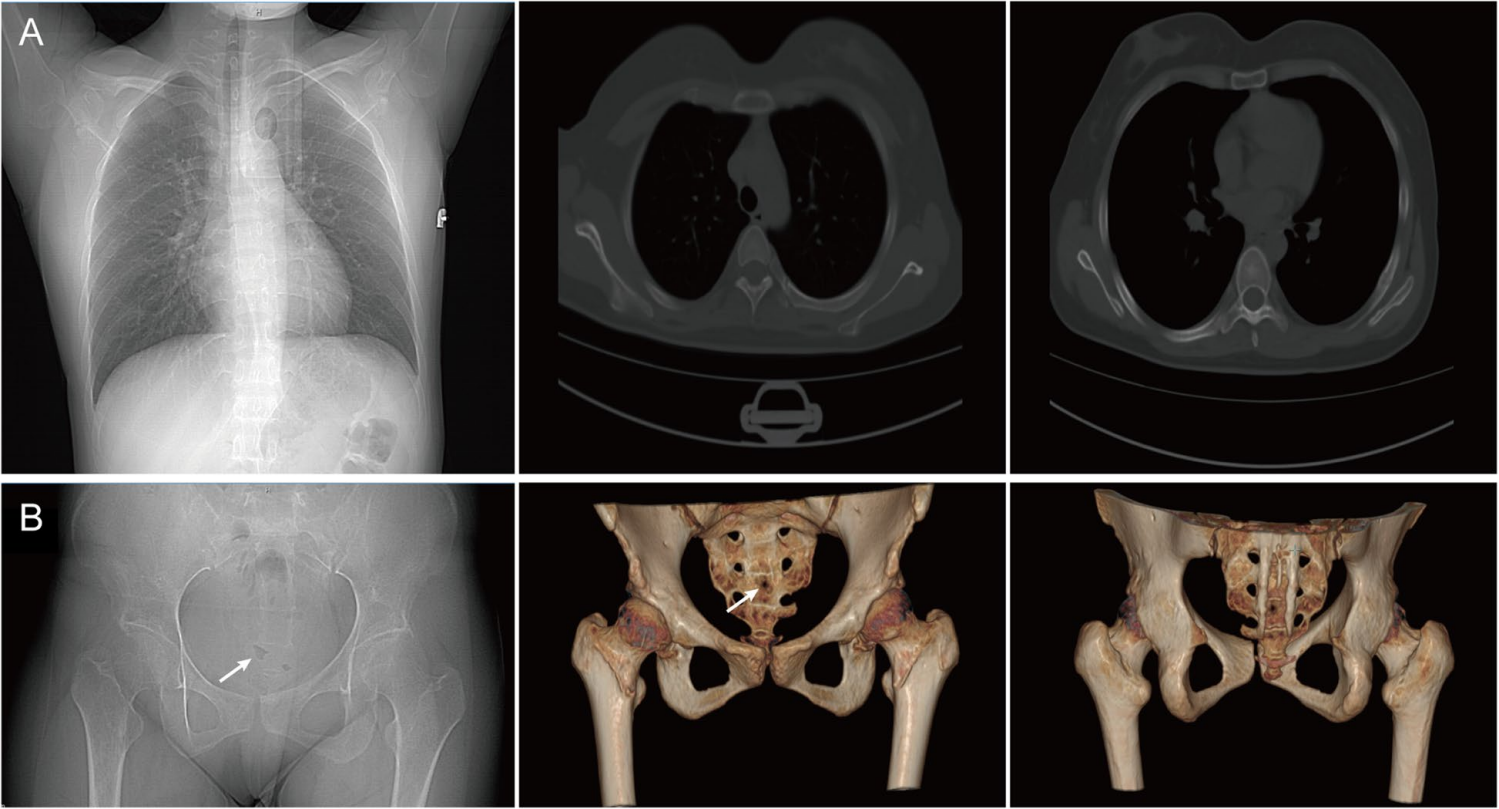
CT imaging of this patient. **A** Chest CT shows bone structure disorder in multiple ribs on both sides. (**B**) Hip joint CT shows nodular high density in the left ilium and sacrum, the bone structure of the remaining two hip joints is intact

Old fractures of multiple ribs on both sides. Left ilium and left sacrum bone islands (Fig. 1).

#### MRI (August 29, 2021)

**Fig. 2:**
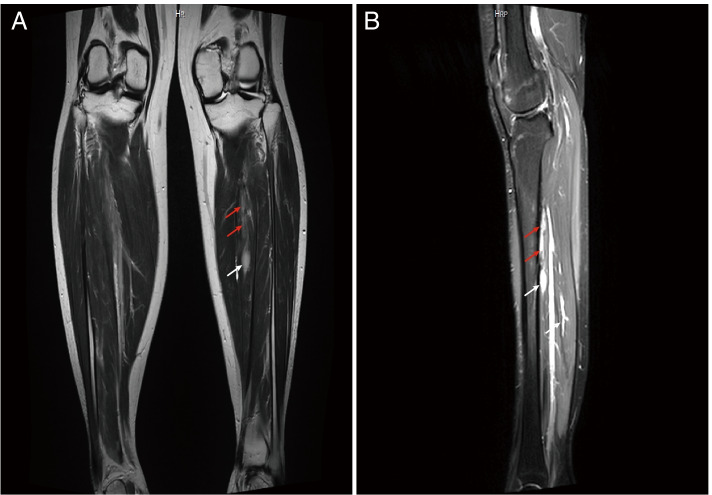
MRI imaging of tumor-induced osteomalacia. MRI shows an elliptic slightly high signal near the cortical bone of the posterior edge of the left middle tibia (1.6*0.7 cm, indicated by the white arrow), and a small nodular, vertical strip abnormal signal outside the bone (1.5 cm, indicated by the red arrows). **A** Coronal MRI, **B** sagittal MRI

An elliptic slightly high signal near the cortical bone of the posterior edge of the left middle tibia (1.6*0.7 cm), and a small nodular, vertical strip abnormal signal outside the bone (1.5 cm) (Fig. 2).

#### DXA (August 12, 2021)

L1-L2 –1.5; L1-L3 –1.5; L1-4 –1.7; L2-L3 –1.7; L2-L4 –1. 8; L3-L4 –1.8; Neck −2.9; Wards triangle −2.7; Great trochanter −2.4; All −2.3.

#### ^99m^Tc-MDP bone scan (August 27, 2021)

**Fig. 3:**
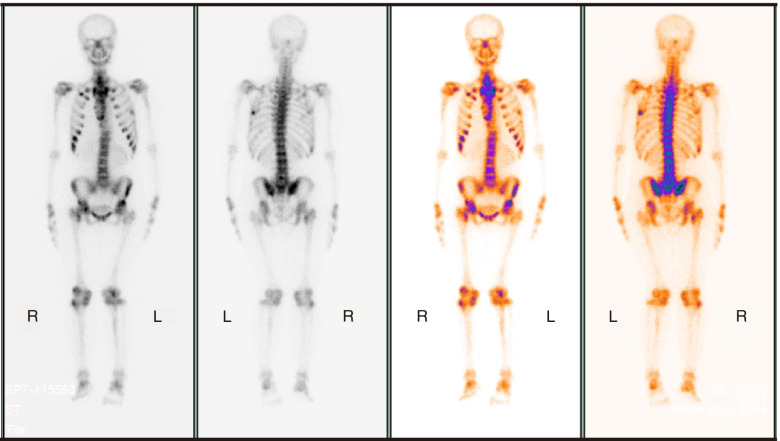
^99m^Tc-MDP whole body bone scan. ^99m^Tc-MDP whole body bone scan shows osteomalacia, with pseudo fractures of multiple ribs and right femoral neck

Osteomalacia, with pseudo fractures of the anterior segment of the right 2nd rib, the axillary segment of the left 7th and 8th ribs, right side of the sacrum, left acetabulum, and right femoral neck (Fig. 3).

#### ^68^Ga-DOTATATE PET/CT (August 27, 2021)

**Fig. 4:**
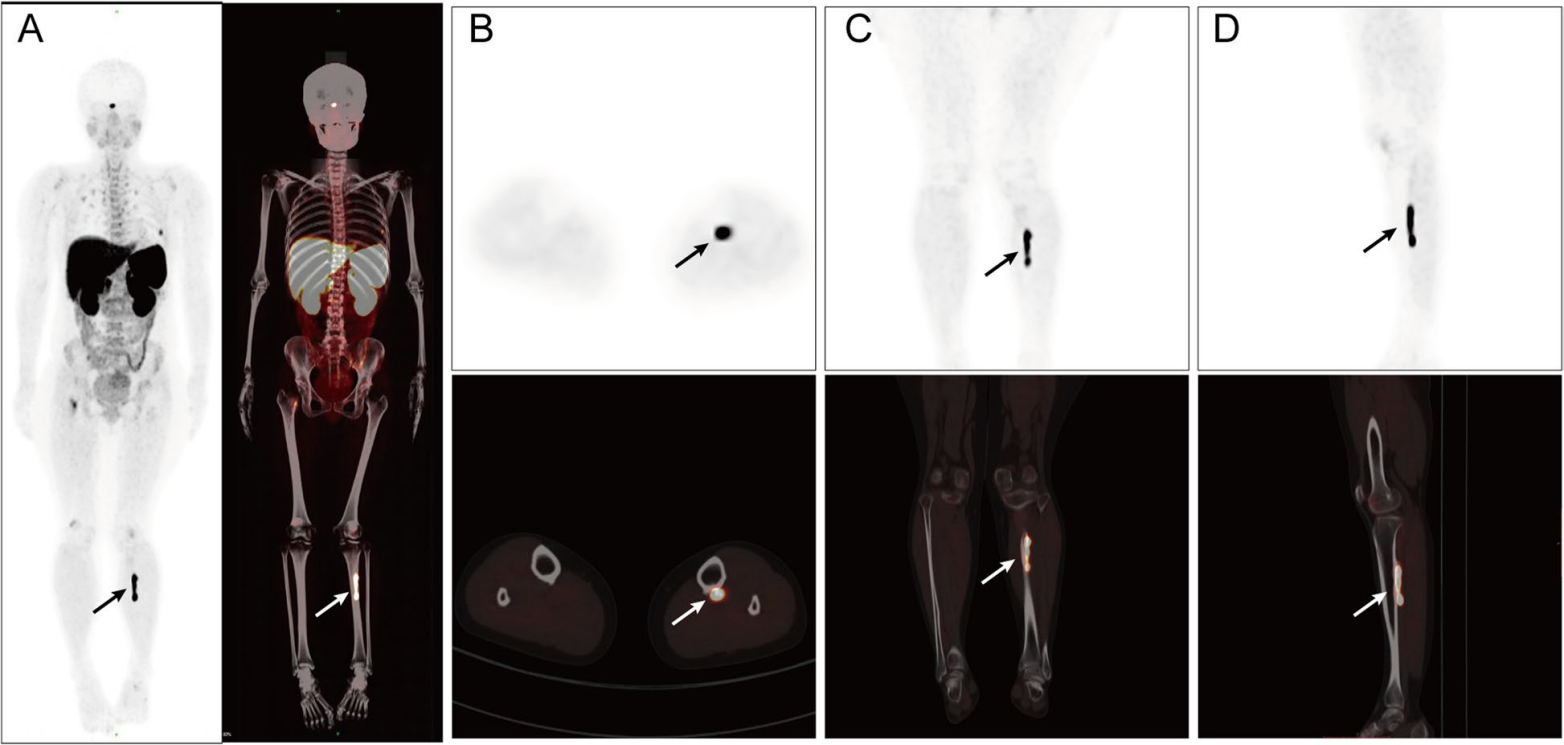
^68^Ga-DOTATATE PET/CT imaging of tumor-induced osteomalacia. ^68^Ga-DOTATATE PET/CT shows abnormal density of left middle and upper tibia with increased SSTR expression. Pseudo fracture of multiple ribs and right femoral neck with increased SSTR expression

Abnormal density of left middle and upper tibia with increased somatostatin receptors (SSTR) expression. Pseudo fracture of multiple ribs (the posterior segment of the left 5th rib, the axillary segment of the left 7th to 8th rib, the anterior segment of the right 2nd rib) and right femoral neck with increased SSTR expression. TIO was considered in the above lesions (Fig. 4).

### Diagnosis and treatment

**Fig. 5:**
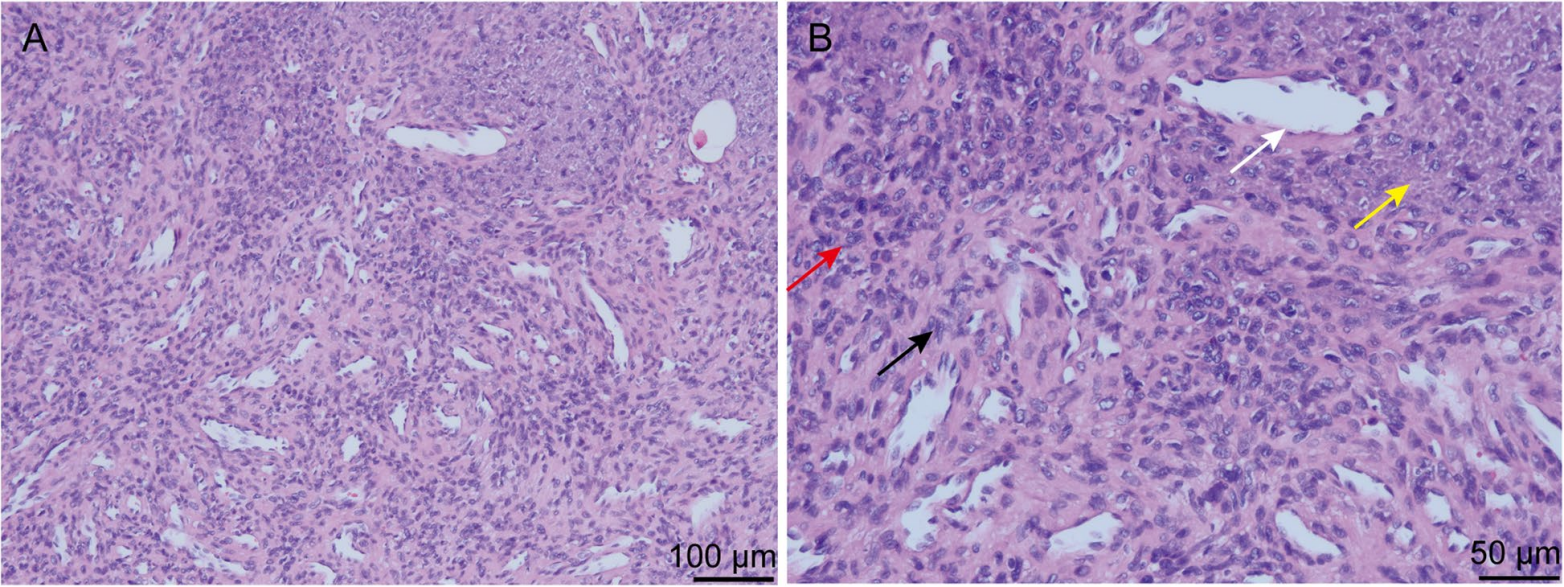
The histological features of phosphaturic mesenchymal tumor (PMT). Low-magnification (**A,** 100×) and high magnification (**B,** 200×) images of a PMT. Highly vascular proliferation (indicated by the white arrow) and spindle-shaped cells (indicated by the black arrow) form most of the background cells and are embedded in a distinctive smudgy matrix. The osteoclast-like multinucleated giant cells (indicated by the red arrow) and “grungy” calcification are scattered in the tumor (indicated by the yellow arrow)

**Fig. 6:**
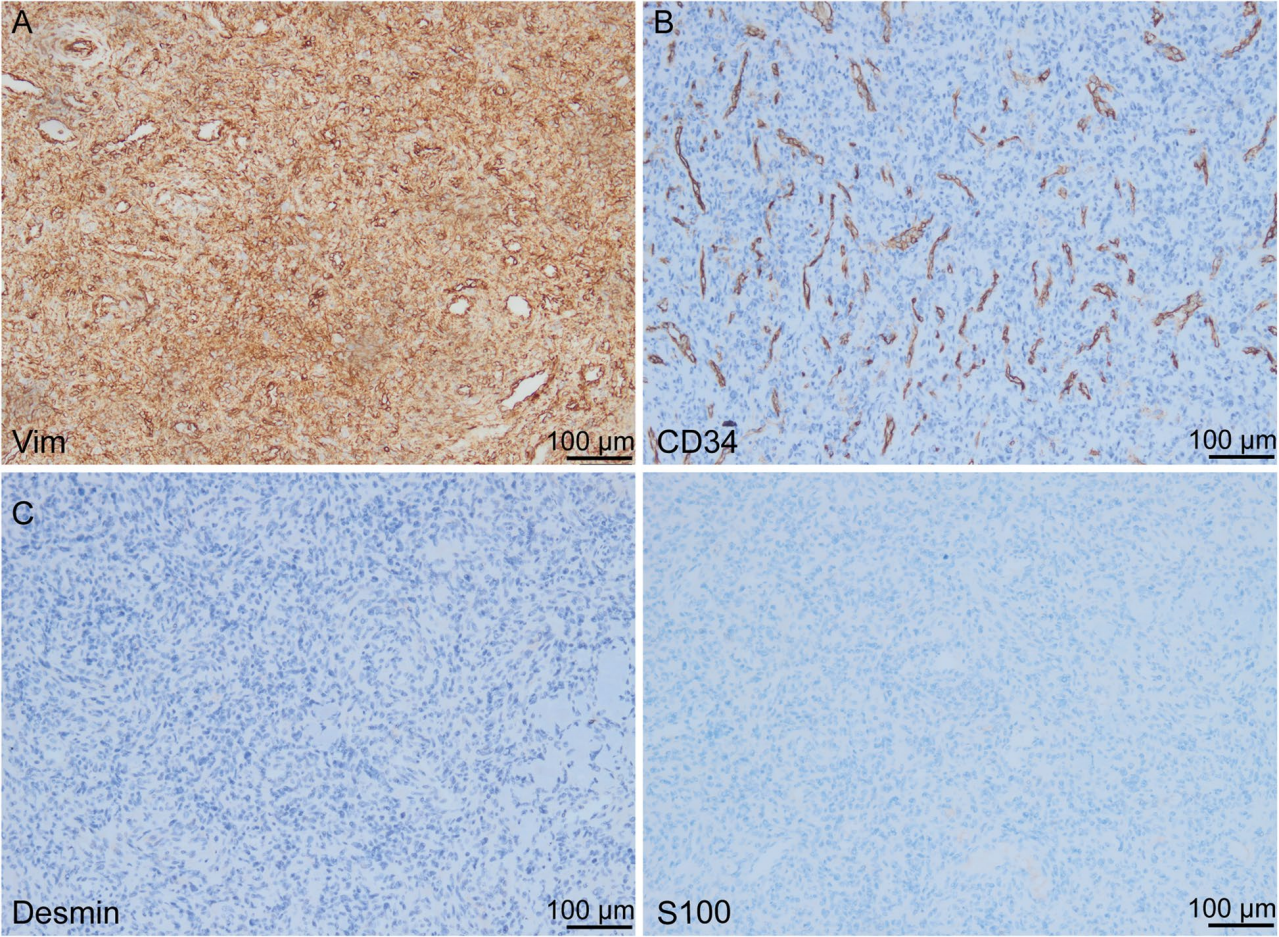
Immunohistochemisty for Vim, CD34, Desmin, and S100 of the tumor (100×). **A** Vim, as a mesenchymal cell marker, is positive in most tumor cells, indicating the tumor originated from mesenchymal tissues (brown). **B** The tumor contains a large number of small capillaries, most of which are positive for CD34 staining (brown) whereas tumor cells are negative. **C** Desmin is characteristically found in muscle cells and associated neoplasms, is negative in PMT. **D** S100 is the specific protein of neural crest-derived tumors, is negative in PMT

**Fig. 7:**
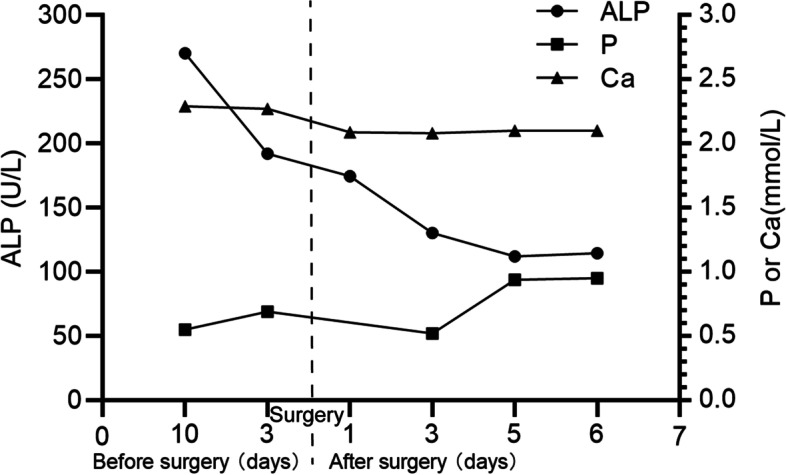
Changes of the serum alkaline phosphatase (ALP), serum phosphate (P), and serum calcium (Ca) before and after surgery. Hypophosphatemia was rapidly improved, and phosphate levels returned to normal within 5 days. However, the elevated serum ALP and the mild hypocalcemia was not improved in the short term

Combined with the above clinical history, physical examination, laboratory evaluation, the patient was initially confirmed to have TIO. The responsible tumor was located in the left proximal tibia. The patient then underwent resection and biopsy of the left tibial tumor on September 6, 2021. During the operation, there was a bony protrusion and bone destruction in the left proximal lateral tibia, and white soft tissue beside the bone. The damaged bone tissue and soft tissue beside the bone were taken and sent for pathological examination. Then we used Kirschner wire to drill holes in the lesion area, and used electric knife to kill the lesion tissue at high temperature. Postoperative histopathological examination demonstrated a spindle cell tumor with prominent vascularity, smoky matrix, “grungy” calcification and a small amount of multinucleated, osteoclast-like giant cells (Fig. 5). Immunohistochemical staining was also performed, and the results are as follows: Vim (+), SATB2 (partly+), CD34 (−), S100 (−), Desmin (−), SMA (−), P53 (−), Ki-67 (positive rate, 1%), STAT6 (−), AE1/AE3 (PCK) (−), compatible with a PMT (Fig. 6). By postoperative day 5, the serum phosphorus normalized (0.94 mmol/L) and symptoms improved (Fig. 7 and Table 3). And now the patient’s systemic bone pain has improved significantly.

**Table 3:** Changes of the serum alkaline phosphatase (ALP), serum phosphate (P), and serum calcium (Ca) before and after surgery

Time (days)	Before surgery		After surgery			
	10d	3d	1d	3d	5d	6d
**ALP (U/L)** (n: 35–100)	270.4	192.1	174.5	130.3	112.1	114.7
**P (mmol/L)** (n: 0.85–1.51)	0.55	0.69	NA	0.52	0.94	0.95
**Ca (mmol/L)** (n: 2.11–2.52)	2.29	2.27	2.09	2.08	2.10	2.10

Hypophosphatemia was rapidly improved, and phosphate levels returned to normal within 5 days. However, the elevated serum ALP and the mild hypocalcemia was not improved in the short term. *NA N*ot available

## Discussion and conclusions

TIO is an acquired metabolic bone disease caused by tumor, and is one of causes of osteomalacia hypophosphorus in adults. Laboratory tests in TIO show refractory hypophosphatemia, elevated alkaline phosphatase, reduced tubular reabsorption of phosphate, low or normal 1,25(OH)_2_D, serum calcium and parathyroid hormone [2, 5]. Most studies believed that TIO is caused by phosphorus-regulating factors secreted by responsible tumors [6]. Among them, FGF23 is the only protein that has been shown to have clinical significance, which results in hypophosphatemia and renal phosphate wasting, reduced 1,25(OH)_2_D synthesis, and osteomalacia [7]. Klotho, as a coreceptor for FGF23, mediates various physiological processes to maintain phosphate and calcium homeostasis. There are different isoforms of Klotho protein: membrane-bound Klotho and soluble Klotho [8]. The ectopic expression of membrane-bound Klotho in PMTs enables a positive feedback loop in FGF23 production via the activation of FGF receptor 1c and exacerbates disease manifestations in TIO [9]. Soluble αKlotho fragment that is produced by ectodomain shedding can circulate in the blood, urine and cerebrospinal fluid [10]. However, the function and mechanism of circulating soluble Klotho in FGF23-producing tumors that cause TIO is yet to be clarified. TIO-associated tumors are usually reported to be PMT that is mostly benign tumors derived from mesenchymal tissue [11]. Histologically, PMT are composed of highly vascular primitive-appearing mesenchymal cells with low nuclear grade and low mitotic activity embedded in a distinctive myxoid to myxochondroid matrix and exhibiting “grungy” or flocculent calcification. Other features include microcystic change, mature fat, chondroid or osteoid-like matrix, and woven bone [12, 13]. The pathological findings of this patient are consistent with this.

Surgery is the only established, definitive treatment of TIO [14, 15]. However, the localization and diagnosis is very difficult because of the small size, hidden location and slow development of tumors, and they may appear in any mesenchymal tissue all over the body, including bone and soft tissue [16]. FGF-23 secreted by tumors is an important pathogenesis of TIO, and serum FGF-23 levels are elevated in the majority of TIO patients. Therefore, systemic segmental venous sampling of FGF-23 facilitates the location of responsible tumors, but it has not been widely used in clinical practice as an invasive examination [17]. Furthermore, serum Klotho measurement maybe explain if the FGF23 cause is independent or dependent on Klotho. Noninvasive imaging examination plays an increasingly prominent role in the localization of TIO. X-ray manifestations of TIO are not significantly different from other osteomalacia, which are typically characterized by blurred trabeculae, multiple false fracture lines or fractures, “double concave” changes in the vertebral body and “triangular” changes in the pelvis [18]. CT and MRI can find hidden fracture and intraosseous edema, and make suggestive diagnosis for adult hypophosphorus osteomalacia patients who have ruled out other causes, but lack specificity [19, 20]. Besides, the specificity and sensitivity of fluorodeoxyglucose-positron emission tomography-computed tomography (^18^F-FDG PET/CT) is also poor because PMT is mostly benign with low metabolic activity [21].

Since most responsible tumors are mesenchymal tissue derived and express the SSTR, SSTR-based functional PET/CT has been used successfully to detect TIO tumors. ^111^In-pentetreotide or octreotide is the first approved radioactive drug for neuroendocrine tumor (NETs) imaging. Its high affinity for SSTR2 and SSTR5 makes ^111^In-pentetreotide single-photon emission computed tomography (Octreoscan-SPECT/CT) useful for the localized diagnosis of TIO [22, 23]. 1,4,7,10-tetraazacyclododecane-1,4,7,10-tetraacetic acid tyrosine-3-octreotate (DOTATATE) is a radionuclide labeled somatostatin analogue, which is mainly used as PET or PET/CT imaging agent for the location diagnosis of SSTR positive NETs. ^68^Ga-DOTATATE, like octreotide, is an antagonist of the SSTR, which upon receptor binding is internalized resulting in accumulation of radioactivity in tumor cells [24]. ^68^Ga-DOTATATE PET/CT has been proved to be superior to Octreoscan-SPECT/CT for possible reasons: (1) ^68^Ga-DOTATATE has higher affinity for SSTR2 and SSTR5 than octreotide [25, 26]; (2) PET/CT provides better spatial resolution than SPECT/CT [23, 27]. In addition, ^68^Ga-DOTATATE imaging has advantages in terms of shorter acquisition time and lower radiation exposure because of its short half-life of 68 minutes, compared with 2.8 days for ^111^In, the isotope used in Octreoscan-SPECT/CT imaging. ^68^GA-DOTATATE PET/CT maximum standardized uptake value (SUV max) also correlates with the clinical and pathologic features of NETs and is an accurate prognostic index [28]. In this case, ^68^Ga-DOTATATE PET/CT demonstrated abnormal density of left middle and upper tibia with increased SSTR expression, thus successfully locating the tumor.

At present, TIO is mostly reported to start in middle age. However, the onset age of this case was prepubescent (12 years old), which is obviously younger than majority of reported cases. Thus it needs to be differentiated from hereditary hypophosphatemia, mainly including: (1) X-linked hypophosphacemic rickets (XLHR); (2) autosomal dominant hypophosphatemic rickets (ADHR); (3) autosomal-recessive hypophosphatemic rickets (ARHR) [29]. Although ADHR, ARHR, and XLHR are biochemically indistinguishable from TIO, they are the hereditary disease caused by gene mutation, often have a family history [30]. Whereas TIO is a paraneoplastic syndrome caused by tumors, and there are responsible tumors in vivo. A definitive diagnosis can be made by genetic testing when necessary. TIO also needs to be differentiated from primary hyperparathyroidism, primary osteoporosis, multiple myeloma, symptomatic Tarlov cysts, Fanconi’s syndrome, and drug-induced hypophosphorous osteomalacia [2].

This patient was diagnosed with PCOS 3 years ago, while sex hormones returned to normal 5 days after surgery. It indicates the possible association between PCOS and TIO. However, as far as we know, its specific mechanism remains to be elucidated. Previous studies have found that 1,25(OH)_2_D stimulates aromatase activity [31, 32], down-regulates estrogen receptor abundance and suppresses estrogen effect in human breast cancer cells [33], and up-regulates androgen receptor gene expression in prostate cancer cells [34]. Thus, vitamin D may play a role in the pathophysiologic mechanism of PCOS by influencing the balance between androgens with estrogens. Another possible pathway is apoptosis, 1,25(OH)_2_D and calcium may be necessary for normal ovarian follicular formation. Loss of apoptotic mechanisms or overexpression of anti-apoptotic factors may lead to the appearance of polycystic ovary [35, 36]. In addition, in women with PCOS, serum levels of phosphorus were found to be negatively correlated with insulin resistance [37].

Neutral phosphorus solution and 1,25(OH)_2_D replacement therapy can be given when TIO responsible tumors cannot be localized or completely resected. Serum phosphorus and symptoms can be improved to some extent, but cannot be cured [6]. The use of octreotide in TIO patients has been abandoned, as modulation of the somatostatin receptor using this somatostatin analogue has not been shown to affect the tumor mass or the phosphate metabolism [38, 39]. FGF-23 secreted by responsible tumors is the key hormone regulating renal phosphorus transport and bone mineralization. Inhibition of FGF-23 is an emerging treatment of unresectable TIO, such as FGF-23 antibodies, FGF-receptor inhibitors and mitogen-activated protein kinase (MAPK) inhibitors [40, 41]. However, whether or not these drugs, of which several are in development, will be safe and effective treatments for TIO remains to be seen, but they clearly hold promise.

TIO, as a rare paraneoplastic syndrome, is easy to be misdiagnosed. When patients develop unexplained progressive multiple bone pain, muscle weakness, hypophosphatemia and elevated alkaline phosphatase, the possibility of TIO should be considered. ^68^Ga-DOTATATE PET/CT is recommended for the location of responsible tumors. Surgical removal and close follow-up are essential for the treatment of TIO. Patients unable to undergo surgery can be treated with neutral phosphorus solution and 1,25(OH)_2_D.

## Data Availability

The datasets used and/or analysed during the current study are available from the corresponding author on reasonable request.

## Abbreviations

TIO: Tumor-related osteomalacia
PMT: Phosphaturia stromal tumor
FGF23: Fibroblast growth factor 23
DXA: Dual-energy X-ray absorptiometry
PCOS: Polycystic ovary syndrome
PINP: Procollagen I N terminal peptide
CT: Computed tomography
MRI: Magnetic resonance imaging
^99m^Tc-MDP: ^99m^Tc-methylene diphosphonate
DOTATATE: 1,4,7,10-tetraazacyclododecane-1,4,7,10-tetraacetic acid tyrosine-3-octreotate
SSTR: Somatostatin receptors
SPECT/CT: Single-photon emission computed tomography
^18^F FDG-PET/CT: Fluorodeoxyglucose-positron emission tomography-computed tomography
ALP: Alkaline phosphatase
NETs: Neuroendocrine tumor
SUV max: Maximum standardized uptake value
XLHR: X-linked hypophosphacemic rickets
ADHR: Autosomal dominant hypophosphatemic rickets
ARHR: Autosomal-recessive hypophosphatemic rickets
MAPK: Mitogen-activated protein kinase
